# Training State-of-the-Art Deep Learning Algorithms with Visible and Extended Near-Infrared Multispectral Images of Skin Lesions for the Improvement of Skin Cancer Diagnosis

**DOI:** 10.3390/diagnostics15030355

**Published:** 2025-02-03

**Authors:** Laura Rey-Barroso, Meritxell Vilaseca, Santiago Royo, Fernando Díaz-Doutón, Ilze Lihacova, Andrey Bondarenko, Francisco J. Burgos-Fernández

**Affiliations:** 1 Centre for Sensors, Instruments and Systems Development, Universitat Politècnica de Catalunya, 08222 Terrassa, Spain; meritxell.vilaseca@upc.edu (M.V.); santiago.royo@upc.edu (S.R.); fernando.diaz-douton@upc.edu (F.D.-D.); francisco.javier.burgos@upc.edu (F.J.B.-F.); 2 Institute of Atomic Physics and Spectroscopy, University of Latvia, 1004 Riga, Latvia; ilzelihacova@gmail.com; 3 Faculty of Computer Science and Information Technology, Riga Technical University, 1048 Riga, Latvia; bondtnt@gmail.com

**Keywords:** skin cancer, multispectral imaging, deep learning, non-invasive diagnosis, melanoma, convolutional neural network

## Abstract

An estimated 60,000 people die annually from skin cancer, predominantly melanoma. The diagnosis of skin lesions primarily relies on visual inspection, but around half of lesions pose diagnostic challenges, often necessitating a biopsy. Non-invasive detection methods like Computer-Aided Diagnosis (CAD) using Deep Learning (DL) are becoming more prominent. This study focuses on the use of multispectral (MS) imaging to improve skin lesion classification of DL models. We trained two convolutional neural networks (CNNs)—a simple CNN with six two-dimensional (2D) convolutional layers and a custom VGG-16 model with three-dimensional (3D) convolutional layers—using a dataset of MS images. The dataset included spectral cubes from 327 nevi, 112 melanomas, and 70 basal cell carcinomas (BCCs). We compared the performance of the CNNs trained with full spectral cubes versus using only three spectral bands closest to RGB wavelengths. The custom VGG-16 model achieved a classification accuracy of 71% with full spectral cubes and 45% with RGB-simulated images. The simple CNN achieved an accuracy of 83% with full spectral cubes and 36% with RGB-simulated images, demonstrating the added value of spectral information. These results confirm that MS imaging provides complementary information beyond traditional RGB images, contributing to improved classification performance. Although the dataset size remains a limitation, the findings indicate that MS imaging has significant potential for enhancing skin lesion diagnosis, paving the way for further advancements as larger datasets become available.

## 1. Introduction

The use of learning algorithms for Computer-Aided Diagnosis (CAD) in the field of medical imaging is becoming increasingly more popular. Dermatologists are already taking advantage of CAD to complement their diagnostic skills. But there is still a complex problem to solve: the automated and non-invasive etymological classification of skin lesions. To date, the only way to unequivocally determine the etiology of a lesion is a biopsy. Under the first-response gold-standard step in diagnosis, which is visual inspection to outline the warning signs of malignancy (asymmetry, border irregularity, changes in color, and changes in diameter and evolution) [1], approximately 50% of lesions appear as equivocal to expert physicians. Consequently, histological examination remains the definitive standard for lesion classification, which is both time-consuming and costly [2].

This is a significant problem due to the progressive trend in the rising incidence and mortality of skin cancer, with an estimated 60,000 people dying annually, 48,000 from melanoma (MM) and 12,000 from other non-melanocytic skin cancers [3]. Melanoma is the most aggressive form, originating from pigment-making cells called melanocytes, and is likely to grow and spread if left untreated, having a five-year survival rate if not treated early [4]. Non-melanoma skin cancers, including Basal Cell Carcinomas (BCCs) and Squamous Cell Carcinomas (SCCs), grow more slowly and usually do not spread. BCCs are the most common type (8 out of 10), while SCCs are more likely to grow into deeper layers of skin [5]. The prognosis for non-melanocytic types of cancer is excellent; however, if left untreated, they can result in significant morbidity and cosmetic disfigurement.

## 2. Recent Literature

Authors prioritize the detection of melanoma, and more specifically its distinction from other pigmented lesions, such as the melanocytic nevus [6], a benign (not cancerous) growth on the skin that is formed of a cluster of melanocytes. They sometimes present a very similar appearance and attributes, so they are particularly hard to differentiate for physicians. A second type of benign lesions, Seborrheic Keratoses (SK), are often included in the lesion classification challenges [7,8,9,10]; BCCs are usually included as well [11,12,13,14].

To address the non-invasive detection of skin cancer, CAD algorithms, based on Deep Learning (DL) architectures such as generative adversarial networks (GANs) or convolutional neural networks (CNNs), are gaining prominence [6]. CNNs have proven to be highly effective in medical image analysis due to their ability to perform convolutions on input images and extracting spatial features for image recognition [15]. Some are designed to take care of the image pre-processing steps like digital hair removal and segmentation from the surrounding skin [16]; others contribute to the automated feature extraction and subsequent classification with a Fully Connected (FC) layer [7,17,18,19] or with a combination of traditional ML classifiers such as Support Vector Machines (SVM), k-Nearest Neighbor (KNN), and Random Forest (RF) [8,20,21]. Several advanced CNN architectures have been developed and adapted for skin lesion classification. For instance, architectures such as Inception, VGGNet, and ResNet have been employed with success in various studies [22,23,24,25]. Some researchers have customized these architectures or designed new ensembles of models to improve classification accuracy [8,9,26,27,28].

The majority of works that train CNNs to perform lesion classification use high-quality medical Red-Green-Blue (RGB) images, like the ones provided by large data repositories such as ISIC with 71,023 dermoscopic images [29], PH2 [16], DermQuest, DermIS [30], HAM10000 [31], etc. These repositories contain vast amounts of data that help train DL models robustly. However, despite achieving very high classification accuracies (Xception trained with HAM10000: 90.48% [32], VGG hybrid trained with PH2: 93.3% [33], and NASNet trained with ISIC 2020: 97.7% [34]), they are not good enough to replace the gold standard. In Table 1, there are more details about some of these works from the recent literature.

Therefore, it is important to exploit other imaging modalities that can provide more information [37], such as confocal laser scanning microscopy (CLSM) [38] and optical coherence tomography (OCT) [35] regarding tissue structure and multispectral (MS) or hyperspectral (HS) imaging [39,40,41] regarding substance content.

MS imaging provides images of skin lesions through several spectral bands with high spatial resolution, capturing pixel-wise spectral features that reflect the absorption properties of skin substances like hemoglobin, melanin, bilirubin, and tissue oxygenation [42,43,44,45,46]. Previous studies have shown that MS imaging can be useful in distinguishing different types of skin lesions, for example, malignant melanoma from benign melanocytic nevi, which often present similar visual characteristics [7,47]. Despite the limited number of MS images available, these studies indicate that incorporating spectral information can improve the performance of DL models in skin lesion classification tasks (RetinaNet trained with 35 SWIR images: 68.8% sensitivity over melanoma [48]; custom model trained with 1304 MS images: 72.0% sensitivity over melanoma [39]; ResNet trained with 76 hyperspectral images: 50.0% sensitivity over melanoma and 88.0% sensitivity over other malignant lesions [49]). In Table 2, there are all of the details about these works from the recent literature.

In this study, we trained two CNNs—a simple CNN and a custom VGG-16 model—using a dataset of MS images that included nevi, melanomas, and BCCs. Additionally, we trained the CNNs using only three images from the MS set—the ones closest to the RGB spectral bands traditionally used in color cameras—to compare classification performance when training with RGB versus MS information. By doing so, we achieved significantly higher precision in classification for both models when trained with the full MS cubes, thereby demonstrating the added value of spectral information over conventional RGB imaging. Currently, algorithms are trained with collaborative RGB datasets, and their accuracy plateaued at around 98%. Hence, the findings in this work provide valuable insights into improving the precision of DL systems for skin lesion classification.

## 3. Methods

### 3.1. Multispectral System and Dataset

**Figure 1:**
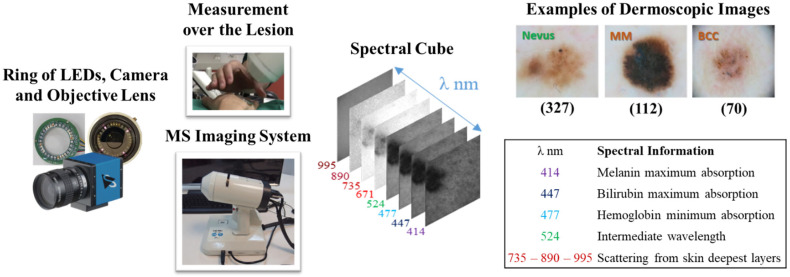
From left to right: ring of LEDs, camera, and objective lens; external view of the MS system; spectral cube of a lesion; 8 spectral wavelengths included in the MS system and corresponding information on the absorption of skin chromophores, and examples of dermoscopic images out of the three lesion classes.

Chromophores in the skin, such as melanin, hemoglobin, water, beta-carotene, collagen, and bilirubin, differ among skin lesions of different etiologies. MS imaging systems can sequentially illuminate skin with light at different spectral bands and collect the reflected light that contains information on these chromophores. In a previous work [45], we developed an LED-based, low-cost, and portable MS system that was able to capture reflectance and color features in the VIS-NIR range. The camera integrated in the head of the hand-held instrument is a CCD sensor with 12-bit depth and resolution of 1280 pixels × 960 pixels (Sony ICX445ALA, Sony Corporation, Tokio, Japan). A lens is coupled to the camera to focus the skin at 40 mm with a field of view of 15 mm × 20 mm (Cinegon, Schneider-Kreuznach, Bad Kreuznach, Germany). A ring of LEDs with peaks at eight different spectral bands (414, 447, 477, 524, 671, 735, 890, and 995 nm) was used to illuminate the skin and obtain a spectral cube for each analyzed lesion. A couple of crossed polarizers were incorporated to eliminate specular reflection from the sweat and grease of the skin. With this device, the spectral cubes of +700 equivocal lesions were acquired from patients that attended the outcare patient clinic in the Melanoma Unit of the Dermatology Department from the Hospital Clinic i Provincial de Barcelona (Spain) and the Skin Cancer Unit from the Policlinico di Modena-University of Modena and Reggio Emilia (Italy). Some spectral cubes had to be discarded due to lack of pigmentation of the lesion, a greater size than the field of view of the lesion, motion artifacts, or hair growth. Finally, the number of lesions from each etiology that were included were 592, from which 332 (56.1%) corresponded to nevi (melanocytic, dysplastic, blue, junctional, and Spitz nevi), 112 (18.6%) were MMs (including MM in situ and lentigo), 70 (11.6%) were BCCs, 33 (5.4%) were SK, 43 (7.1%) were other benign lesions, such as, angiomas, dermatofibromas, and actinic keratosis, and 7 (1.2%) corresponded to SCCs. In order to be left with a comparable and sufficiently high number of lesions for each class, nevus (327), MM (112), and BCC (70) were the three classes used to feed the DL architectures. The MS system, spectral cube content, and samples of dermoscopic images out of the three lesion classes are illustrated in Figure 1. The diagnosis of the different lesions in the dataset stated by physicians in the dataset are presented in Table 3.

All patients provided written informed consent before any examination, and ethical committee approval was obtained. This study complies with the tenets of the 1975 Declaration of Helsinki (Tokyo revision, 2004). The lesions were diagnosed by dermatologists (S.P., J.M., and G.P.) using a commercial dermoscope and the confocal laser scanning microscope VivaScope^®^ 1500 (MAVIG GmbH, Munich, Germany). When malignancy was suspected, the lesion was excised and a histological analysis was carried out.

### 3.2. Data Preprocessing and Augmentation

The dataset was highly imbalanced, even down to the three main classes (nevi, MM, and BCC), having a ratio of 4:1 for nevi images to images containing malignant lesions. The authors resolved this issue by either undersampling, selectively performing data augmentation over some classes, or by weighting the loss function during training [7,47]. We selected a custom function [50] to generate class weights for weighting the loss, detailed in Section 3.3. Nevertheless, this imbalance was still likely to produce bias in the learning models, so a balanced subset of the training and test data were created by downsampling the data to 70 nevi, 70 melanomas, and 70 BCC spectral cubes.

The images in the spectral cubes were not preprocessed to become normalized to zero-mean and unit variance, since the images had been previously calibrated (Equation (1)) with a standard neutral gray sample (Neutral 5, X-Rite ColorChecker^®^, X-Rite Inc., Grand Rapids, MI, USA) and with images captured in the dark to compensate for straylight noise, providing high-contrast images with normalized values (reflectance images).

(1) $$R_{Lesion}^{\lambda }(i,j)=k\frac{S_{Lesion}^{\lambda }(i,j)-S_{Dark}^{\lambda }\left(i,j\right)}{S_{Neutral}^{\lambda }(i,j)-S_{Dark}^{\lambda }\left(i,j\right)}$$

where ${R^{\lambda }}_{Lesion}\left(i,j\right)$ is the reflectance value of each pixel $\left(i,j\right)$ on the images taken at each spectral band λ, calculated as the spectral value ${S^{\lambda }}_{Lesion}\left(i,j\right)$ for each pixel on every spectral image λ, calibrated with ${S^{\lambda }}_{Dark}\left(i,j\right)$ and ${S^{\lambda }}_{Neutral}\left(i,j\right)$, which are the intensity of the pixels of the $\;\lambda_{th}$ image taken in the dark and of the $\lambda_{th}$ image of the neutral gray sample, respectively; and k is the calibrated reflectance of the neutral gray sample measured with a spectrometer. All images $S_{Lesion}^{\lambda }(i,j)$ were taken with the same settings and fixed position.

The images in the reflectance cubes were formatted appropriately to be squared images of resolution 128 pixels × 128 pixels as an input into the neural networks. Bigger image resolutions were tested, but no improvement in the accuracy over the inference was obtained; rather, improvements out of memory errors were retrieved when the resolution was too big. Lesions were not segmented from background healthy skin. No further preprocessing steps were required, like digital hair removal or artifact correction, since images of this kind were discarded in the previous steps by visual inspection.

**Figure 2:**
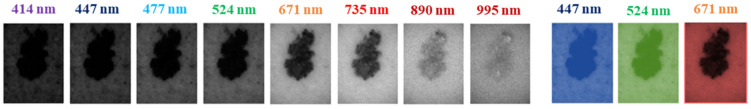
Reflectance images of a lesion taken at λ = 414–995 nm and reflectance images closer to RGB channels.

In some training instances, an additional preprocessing step was implemented to choose only three images from the reflectance cube at the spectral bands 447, 524, and 671 nm, which were the ones closer to the peak wavelengths of the RGB channels of a conventional camera. In Figure 2, there is the full MS set of images and these three images.

The balanced training dataset contained a total of only 210 images, so a robust data augmentation scheme was implemented. This scheme included rotations and random flips applied to the training set. Keras’ experimental preprocessing layers were used to construct the aforementioned data augmentation pipeline. A layer for image augmentation was added after the input layer within the Deep Learning architectures to facilitate synchronous training on the GPU. Approximately, the total number of reflectance cubes was augmented by a factor of 50.

### 3.3. Training Strategy Selection

To select the training strategy, we launched different training instances on four models using the reflectance cubes containing the eight images of normalized values described in Section 3.2.

To train our models, in all instances we chose a training scheme commonly used in the literature which involved dividing our data into training/validation and test sets with an 80–20% ratio. The training/validation set (80%) was varied, swapping images between training and validation using a 5-fold cross-validation scheme, with a proportional number of samples from each class using the Scikit Learn StratifiedKFold class. The remaining set (20%) was used to test how well the models could classify unseen lesion images.

Initially, we trained with the entire dataset and, since it was highly imbalanced, a custom function [50] based on Python’s compute_class_weight was used to generate class weights (Equation (3)) for weighting the loss function (Equation (2)) during training.

(2) $$CE=-\sum_{i\in C}t_{i}\log\left(s_{i}\right)$$

where $t_{i}$ and $s_{i}$ are the ground truth and the model score for each class i in C, respectively. Softmax activation function is applied to the scores before the categorical cross-entropy (CE) computation. The loss weighting function was also applied to categorical CE when using the whole unbalanced dataset. The weights for the loss function are calculated as shown below:

(3) $$w_{i}=-\sum_{j=1}^{m}Y_{i}$$

where $Y_{ij}$ is the value of class i in example j and m is the number of examples in the original training set. Since $Y_{j}$ is a one-hot vector, the value of $Y_{ij}$ is either 0 or 1. The mean of this along the number of examples gives the frequency of class i in the dataset.

Also, a balanced subset was created by downsampling the dataset. Despite containing a total of only 210 images (70 nevi, 70 MMs, and 70 BCCs), the models used to test the different training strategies showed a better performance when trained with the proportional sample set.

Lastly, two types of training instances were conducted. In the first type, the model was declared and trained from scratch every time a new fold of data was used in cross-validation. In the second type of instance, the model was further trained with every fold of new data. We found that the performance of the models trained with the second type of instance was better.

The models used for testing were four different CNN architectures: a simple CNN with six two-dimensional (2D) convolutional layers, a custom VGG-16 model with three-dimensional (3D) convolutional layers, a custom ResNet-34 that also performs 3D convolutions to extract features from the whole reflectance cube at once, and ResNet50 imported from Keras applications [51] with 2D convolutional layers.

The models were trained from scratch with the dataset of reflectance cubes. Transfer learning was not considered (with models trained with datasets such as ImageNet [52]), as it is not recommended when working with images with very different attributes than the objects of the real world, in addition to a very high intra-class variance and very low inter-class variance [12,27,53,54].

**Figure 3:**
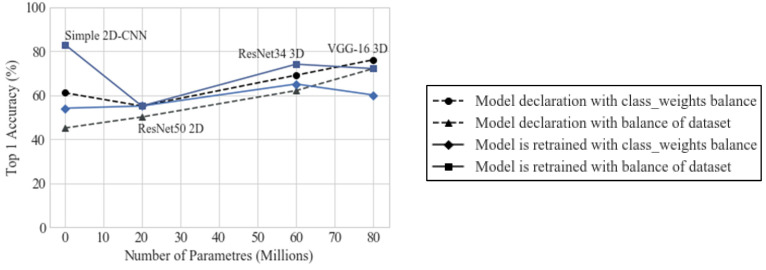
Top 1 accuracy (%) obtained with the models when trained with the different strategies.

Figure 3 shows the performance of these models that were trained with the different strategies explained here.

It was determined that the best strategy was to use the balanced set and further train the model with every fold of data, and that the CNNs that achieved the best performance in terms of top 1 accuracy and the most consistent results for different training instances were the simplest CNN and the VGG-16 model. Table 4 show the performance of these models with the selected training strategy.

The simplest CNN and the VGG-16 model were trained with only three images from the reflectance cube, the ones closest to the wavelengths of the images that would be obtained with an RGB camera.

All instances were launched with a random set of 210 reflectance cubes extracted from the total dataset, and the hyperparameters were varied following a random grid search strategy [55] to try to achieve the highest performance: batch sizes from 2 to 10 and learning rates (LR) from 10^−5^ to 10^−6^. One hundred epochs were set for every instance.

### 3.4. Deep Learning Architectures

Two architectures were chosen for our study: a simple CNN with six 2D convolutional layers, and a custom VGG-16 model with 3D convolutional layers.

**Figure 4:**
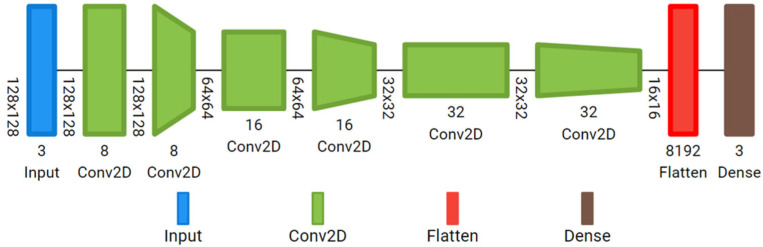
Custom 2D-CNN with six 2D convolutional layers.

Simple custom 2D-CNN: It has an input layer ready to accept the reflectance cube (128, 128, 8), followed by six convolutional layers with ReLu activation; 8, 16 and 32 filters, with kernel size 3; stride 1 in the odd layers, and stride 2 in the even layers. In this case, the features in the reflectance cube are extracted from each individual image, not from the whole cube at once. It has a final dense layer with Softmax activation. The total number of trainable parameters of the small customized 2D-CNN is 43,123. The model is represented in Figure 4.

**Figure 5:**
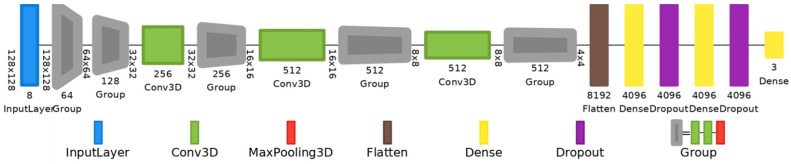
VGG-16 model with 3D convolutional layers, 3D max-pooling layers, and dropout regularization.

Customized VGG-16 with 3D convolutions: It has thirteen 3D convolutional layers, five 3D max pooling layers, and three dense layers. The number of filters is 64 (from the 1st to 2nd convolutional layers); 128 (from the 3rd to 4th); 256 (from the 5th to 7th); 256 (from the 8th to 10th); and 512 (from the 11th to 13th); kernel size 3, stride set to 1, and kernel initialization type He uniform. They all have ReLu activation. Softmax activation is selected in the output layer. To prevent overfitting, a value of 0.5 was used for a dropout regularization in the fully connected layers. The total number of trainable parameters of the customized VGG-16 is 80,328,515. The full architecture is depicted in Figure 5.

Both were trained with reflectance cubes containing the eight images and later with reflectance cubes containing only three images, simulating RGB bands.

### 3.5. Performance Metrics

The metrics to evaluate the classification performance of the two models were the following:

Confusion matrix: Three-class CM. Each entry denotes the number of predictions made by the model, where it classified the classes correctly or incorrectly. It was customized as a Keras metric to monitor results during validation and the final classification performance during testing. TP: true positives; TN: true negatives; FP: false positives; FN: false negatives.

(4) $$CM=\left(\begin{array}{ccc} Class\;1 & Class\;2 & Class\;3\\ c_{11} & c_{12} & c_{13}\\ c_{21} & c_{22} & c_{23}\\ c_{31} & c_{32} & c_{33} \end{array}\right)\;\;\;\;\;\;\;\;\begin{array}{c} TN\\ \left(c_{22}+c_{33}\right)\\ \left(c_{11}+c_{33}\right)\\ \left(c_{11}+c_{22}\right) \end{array}\begin{array}{c} FN\\ \left(c_{12}+c_{13}\right)\\ \left(c_{21}+c_{23}\right)\\ \left(c_{31}+c_{32}\right) \end{array}\;TP\;\;\;\;\;{\;c}_{11}\;\;\;\;\;\;\;\;c_{22}\;\;\;\;\;\;\;{\;c}_{33}\;FP\;{\;\;\;\;(c}_{21}+c_{31})\;\;\;\;{(c}_{12}+c_{32}){\;\;\;\;(c}_{13}+c_{32})$$

Accuracy: The function categorical accuracy from Keras metrics was selected. The accuracy accounting for final classification performance over the test set was derived from the CM and averaged among the lesion classes.

Sensitivity (SE)/Specificity (SP): TP/TN divided by the total number of lesions for each class.

Precision (P): derived from the CM as TP divided by the total number of positive cases (TP and FP) for each lesion class.

F1 score: the harmonic mean of SE and P, calculated for each of the three classes from the CM.

**Figure 6:**
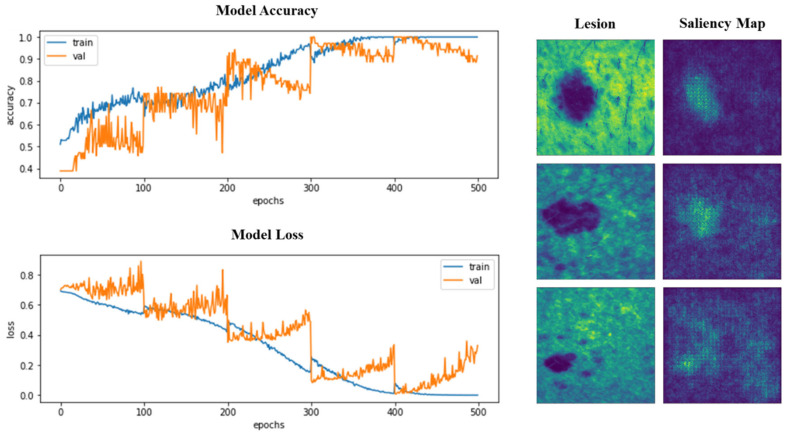
**Left**: Top 1 accuracy and loss during the training of 3D VGG-16, for training and validation sets, split and used in a cross-validation scheme. **Right**: Attention or saliency maps for three different lesions after VGG-16 3D has been trained.

Accuracy and loss were also monitored during training and validation. Similarly, the saliency or attention maps of the models were inspected. In Figure 6, the learning curves and saliency maps for the 3D VGG-16 model are represented.

We were able to verify that, in the last folds, learning was more stable, with less abrupt changes in the graphs, but overlearning began to appear and was more pronounced. In the images of the saliency maps, we could see how the pixels with the highest intensity fall on the area of the lesions, indicating that the attention of the models is focused on the lesions and not particularly on the surrounding skin. Trained models will be made available as Supplementary Materials (GitHub link at the Supplementary Materials Section).

### 3.6. Implementation

A state-of-the-art computer with an AMD Ryzen 9 3950X 16-Core processor (Advanced Micro Devices, Inc., Santa Clara, CA, USA), 64GB of RAM (Kingston Technology Europe Co LLP, Sunbury-on-Thames, UK), and a Quadro RTX 4000 GPU (Nvidia, Santa Clara, CA, USA) was used to implement the trainings. The operative system installed was Windows 10 Pro. Anaconda with Python 3.8.12 and TensorFlow GPU 2.4 release, together with Cuda v. 10.8 libraries, were used to perform training on the GPU. Keras 2.4.3 was used as an API.

## 4. Results

The test set—20% of the images not seen by the models during training—was used to evaluate their classification performance. The classification performance of the 2D-CNN and 3D VGG-16 models was evaluated when trained with all reflectance information versus the classification performance when trained only with images corresponding to the spectral bands 447, 524, and 671 nm. The results (in terms of the metrics described in Section 3.5 are presented in Table 5 below.

It was found that the classification accuracy obtained in the first case was much higher, obtaining 71% for the 3D VGG-16 model and 83% for the 2D-CNN model. In the second type of training instances, 45% was obtained for 3D VGG-16 and 36% for 2D-CNN. Therefore, we can conclude that spectral information does provide something more than just using color or morphological information, so that the training of the CNNs is better.

The best results were retrieved by the simple custom 2D-CNN, but, in general, from Figure 3, it is clear that the model that retrieved the most consistent outcomes is custom 3D VGG-16 model, being the one that has more trainable parameters.

## 5. Discussion

The primary limitation remains the access to a sufficient number of images for training DL models. This challenge is especially pronounced when using images from emerging imaging technologies, such as MS imaging, where, like other researchers, we face constraints due to the limited number of available MS images. These are nowhere near the volume of extensive datasets like ISIC, HAM10000, PH2, etc., which only include color/monochromatic images. Additionally, images acquired through various systems often exhibit significant differences in resolution, contrast, and reference marks, complicating the unification of images and the creation of large public MS image repositories, even though they share the same imaging modality.

Despite the limited dataset size, the results of this study have been remarkably positive. The four models deployed were capable of distinguishing between three different lesion classes, learning these distinctions, and accurately classifying an unseen test set comprising 20% of the data. Beyond demonstrating that MS images are effective for training DL architectures, we confirmed that the spectral information they contain is not made redundant by the data provided by traditional RGB images, which typically teach neural networks to recognize morphology and color. According to our findings, neural networks are also adept at detecting and learning the spectral differences among lesions. In addition to discerning between malignant and benign lesions, they are capable of recognizing variations among different etiologies.

Training with the complete spectral cube, we achieved accuracy rates exceeding 80% for multiclass classification of the three etiologies and nearly 90% for binary classification of malignant (MM and BCC) versus benign (nevus) lesions, proving valuable for clinical screening. Sometimes, as noted by some works in the literature [39], a simpler model may be more effective for classifying a small dataset. It is often best to customize a model or find one completely tailored to your dataset using techniques like Differentiable Architecture Search (DARTS). However, the results obtained with the 3D VGG-16 were generally stable across all instances, likely because this model processes the spectral cube in its entirety, thus correlating significant traits found in one channel with those in others. Some studies [49] have already demonstrated the efficacy of neural networks with 3D convolutions in classifying MS images of skin cancer lesions.

Regarding the selection of hyperparameters, we opted for the grid search method due to its exhaustive and systematic exploration of predefined values, ensuring thorough evaluation and reproducibility. Although alternative optimization methods such as evolutionary strategies, hill-climbing, and Adaptive Particle Swarm Optimization (APSO) have been widely used for hyperparameter tuning in complex problems, they often introduce additional computational overhead and require specialized expertise to fine-tune properly. Given the relatively small dataset and the focus on interpretability, grid search provided a practical balance between computational efficiency and effectiveness.

We found that the best strategy was to train with a balanced sample set and repeatedly train the same model across different folds of data using a cross-validation scheme that divides the training and validation sets into different partitions. The most effective division was into partitions from eight to ten; however, to optimize computing time, we set five folds. Previous studies typically used between five and ten folds. In the future, it might be interesting to test U-Net and its variants to segment the lesions from the surrounding healthy skin as a preliminary step to classification, as performed by previous authors, although this has not been crucial others [39]. Our models’ attention maps (specifically, the 3D VGG-16 model in Figure 6) show hot pixels predominantly over the lesions rather than the surrounding skin.

On the contrary, we discovered that the data augmentation step was particularly critical in the process. Not all types of data augmentation yielded correct results; for instance, random image cropping produced poor outcomes. While altering image intensities did not produce negative results, we opted for caution with this type of augmentation. Even though we performed data augmentation during training, this approach remains debatable, as many suggest that, when a small dataset is available, it is preferable to perform augmentation before training [56] (Neptune.ai). Another option to enhance our results could involve testing this approach. There is also debate about whether to apply augmentation only to the training set or to the validation set as well. Some claim that the best results are achieved by also applying data augmentation to the validation set [57]; however, we could not confirm this in our study. Others argue that this practice is incorrect, since augmentation aims to ensure better model generalization [58,59].

We also believe it is crucial to calibrate images prior to training to ensure normalized gray values across the dataset; if calibration is not performed as a preprocessing step to obtain reflectance images from spectral ones, it is important to standardize images to zero-mean and unit-variance. Contrary to expectations, using a smaller number of lesions by undersampling the dataset to equalize numbers across each class yielded similar accuracy results and improved discrimination of malignant lesions. Using the entire dataset would have made a significant difference only if it substantially increased the number of images (e.g., from 200 melanomas to 2000). Adjusting the loss function does not seem to be a definitive solution; if effective, it would resolve the challenges of large datasets with a significant imbalance in lesion numbers. We observed that changes in image resolution, batch size, or optimizer choice did not enhance classification results; the only critical parameter was the learning rate (LR).

## 6. Conclusions

This study effectively demonstrates the superior capabilities of MS images compared to traditional RGB images in training DL models for skin lesion classification. Our findings emphasize that spectral information not only enhances the accuracy of such models, but also introduces critical non-redundant data closely related to the absorption peaks of tissue chromophores and components, enabling more nuanced distinctions between lesion types.

Our study also highlights the importance of training strategies, particularly the benefits of using a balanced sample set and applying cross-validation with optimal partitions to improve model performance.

Nevertheless, challenges remain, particularly regarding the accessibility and uniformity of MS images needed to train these models effectively. Achieving 100% accuracy remains absolutely crucial. For this reason, state-of-the-art DL algorithms have not yet been fully integrated into healthcare systems. Currently, CAD algorithms, along with emerging imaging modalities, are primarily able to assist professionals in confirming their assessments after visually examining a lesion.

Preferred imaging modalities for dermatological assistance include CLSM and OCT. However, both have limitations. CLSM provides high cellular resolution, but has restricted penetration depth, which can result in false-negative diagnoses for tumors located below the papillary dermis. OCT, on the other hand, offers a resolution that cannot determine cellular types, but is limited to detecting significant structural changes.

Today, DL algorithms are still primarily used in clinical settings for tasks that do not demand perfect precision. For instance, they are trained with electronic medical records to predict in-hospital mortality, optimize hospital management (e.g., reducing patient no-shows), and, in radiology, to detect and segment tumors in images, thereby improving diagnostic accuracy and efficiency.

Our results are a promising step forward, demonstrating that the use of new imaging modalities to train DL algorithms could significantly enhance their classification capabilities, paving the way to achieving non-invasive diagnostic solutions.

In conclusion, this research validates the significant role of spectral information in DL and establishes a benchmark for future studies to build upon.

## Figures and Tables

**Table 1:** State-of-the-art works on training DL architectures for the automated classification of skin lesions. A: atypical; B: benign; C: congenital; H: healthy; M: malignant; MM: melanoma; non-MMC: non-melanocytic cancer; SCC: squamous cell carcinoma; SK: seborrheic keratosis; MLP: multilayer perceptron; WT: wavelet transform; FT: Fourier transform; FF-OCT: Fast Fourier OCT; HSV: Hue Saturation Value; AUC: Area Under the Curve; SE: Sensitivity, SP: Specificity.

	Dataset Samples	Model	Approach	Data Splitting (%)	Accuracy	AUC	SE	SP
**Harangi et al.,** **2018** [9]	ISIC 2017 (2600): MM (491), Nevi (1765), SK (344).	Ensembled CNN out of AlexNet, VGGNet, GoogLeNet.	1. Aggregation of robust CNNs. 2. Feature extraction.	Training, validation (80%). Test (20%).	0.85 MM, 0.88 SK	0.85 MM, 0.93 SK	0.40 MM, 0.71 SK	0.72 MM, 0.85 SK
**Mahbod et al.,** **2018** [8]	ISIC 2016 + 2017 (2787): MM (518), SK (396), B. lesions (2389).	Pre-trained ensembled CNNs.	1. Color normalization + Mean RGB value subtraction + Data augmentation. 2. Ensembled CNNs for feature extraction and SVM as classifier.	Training, validation (80%). Test (20%).	-	0.73 MM, 0.93 SK (for pre-trained) | 0.87 MM, 0.96 SK (for fine-tuned)	-	-
**Mandache et al.,** **2018** [12]	FF-OCT images (40–108,082 patches): BCC (48,970), H. skin (59,112).	VGG-16 and InceptionV3 pre-trained on ImageNet. CNN of 10 layers (trained from scratch).	Feature extraction and classification.	Training, validation (80%): Test (20%).	0.96 (proposed CNN)-0.89 (VGG-16)-0.91 (InceptionV3)	-	-	-
**Refianti** **et al.,** **2019** [18]	ISIC 2017 (198): MM (99), non-MMC (99).	LeNet-5.	1. Data augmentation. 2. Feature extraction with CNN.	Training, validation (80%): Test (20%)	0.95	-	0.91	-
**Saba** **et al.,** **2019** [16]	ISIC 2016 + 2017 + PH2 (4229): B. lesions, Nevi, MM.	InceptionV3.	1. Contrast enhancement + HSV color transformation + lesion boundary extraction (segmentation). 2. Feature extraction with Inception V3	Cross-validation kfold = 20. Training, validation (70%): Test (30%).	0.98 (averaged-PH2), 0.95 (ISIC 2016), 0.95 (ISIC 2017-best)	0.98 (ISIC 2017-best)	0.95 (ISIC 2017-best)	0.98 (ISIC 2017-best)
**Serte** **et al.,** **2019** [10]	ISIC 2017 (2750): MM, SK, M. Nevi.	Pre-trained ensemble of ResNet-18 and ResNet50.	1. Gray-scale transformation. 2. Implementation of wavelet transform (WT = FT) + Data augmentation of MM and SK. 3. Fine-tuning with WT and images + Model fusing.	Training and validation (80%): Test (20%).	0.84 MM, 0.79 SK	-	0.96 MM, 0.81 SK	-
**Adegun** **et al.,** **2020** [28]	ISIC 2017 + PH2 (2860): MM, non-MMC.	Encoder-decoder network.	1. Remove of noise (hair, artifacts) + Zero mean unit variance normalization + Data augmentation. 2. Multi-stage and multi-scale pixel-wise classification of lesions.	Training, validation (80%): Test (20%): 600 ISIC + 60 PH2.	0.92 ISIC, 0.93 PH2	-	-	-
**Maiti** **et al.,** **2020** [19]	ISIC 2017 + MED NODE-(2170): MM (1070), Nevi (1100).	AlexNet, VGG custom CNN.	1. Contrast enhancement + Segmentation. 2. Feature extraction with CNNs.	Training and validation (100%) No test set.	0.72 (AlexNet), 0.68 (VGGNet), 0.97 (custom CNN-best)	-	-	-
**Rodrigues** **et al.,** **2020** [21]	ISIC 2017 + PH2-(1100): MM (174), Nevi (726), MM (40), C. Nevi (80), A. Nevi (80).	Pre-trained VGG, Inception, ResNet, Inception-ResNet, Xception, MobileNet, DenseNet, NASNet.	1. Fine-tuning for feature extraction. 2. Use of classic classifiers: Bayes, MLP, SVM, KNN, and RF. 3. IoT system.	5 instances for each combination of CNN and classifier. Training, validation (90%): Test (10%).	0.97 ISIC, 0.93 PH2 (DenseNet20 and KNN-best)	-	0.97 ISIC, 0.93 PH2	-
**Ho et al.,** **2021** [35]	FF-OCT tomograms-(297–130,383): SCC (43,900), Dysplasia (42,583), H. skin (43,900).	ResNet-18	1. Model training. 2. Heat map extraction.	10-fold cross-validation. Training, validation (85%). Test (15%).	0.81	-	-	-
**Jojoa-Acosta et al.,** **2021** [36]	ISIC 2017-(2742): B. lesions (2220), M. lesions (522).	ResNet152	1. ROI extraction using the Mask and Region-based CNN. 2. Data augmentation balancing lesion ratio. 3. Fine-tuning of ResNet152 for feature extraction and classification.	Training, validation (90%). Test (10%).	0.91	.	0.87 (over MM)	-
**Mendes et al.,** **2021** [14]	MED-NODE + Edinburgh + Atlas-(3816): 12 lesions classes including MM, Nevi, BCC and SCC.	Pre-trained ResNet-152	1. Data augmentation over training and validation. 2. Fine-tuning of the model.	Training, validation (80%). Test (20%).	0.78	-	-	-
**Abbas and Gul,** **2022** [34]	ISIC 2020 (30,000+ images, subset of 4000 images used: Melanoma: 584, Nevus: 2000, Others: 1416).	NASNet (Modified) with global average pooling, fine-tuned.	1. Transfer learning from NASNet pre-trained on ImageNet. 2. Label-preserving augmentations applied (rotation, flipping). 3. Data pre-processing includes ROI cropping and artifact removal.	Training: 75%, Testing: 25%	0.98	-	0.98	0.98

**Table 2:** State-of-the-art works on training DL architectures with MS/HSI imaging datasets for the automated classification of skin lesions. BE: benign epithelial; BM: benign melanocytic; D: dysplastic; H: healthy; HK: hyperkeratotic; non-MMC: non-melanocytic cancer; ME: malignant epithelial; MM: melanoma; MM-like: melanoma-like; PB: pigmented benign; AF: auto-fluorescence; exNIR: extended near-infrared; DARTS: differential architecture search; IR: infrared; SWIR: short-wave infrared; VIS: visible.

Authors	Dataset Samples	MS Sensitivity	Model	Approach	Data Splitting (%)	Accuracy	AUC	SE	SP
**de Lucena et al.,** **2020** [48]	SWIR spectroscopy images (35): MM (12 samples, 34 parts), D. nevi (72 parts), H. skin (17 parts).	IR: 900 nm–2500 nm (256 spectral bands).	RetinaNet (Resnet50 model on backbone).	1. Reduction in the spectral dimension of each SWIR. 2. Feature extraction and classification with RetinaNet.	Training, validation and test.	0.688 MM, 0.725 Nevi	-	-	-
**La Salvia** **et al.,** **2022** [49]	HS images (76–125 bands): MM-like lesions, ME lesions, BM lesions, BE lesions.	VIS-exNIR: 450 nm–950 nm (76 spectral bands).	Pre-trained ResNet-18, ResNet-50, ResNet-101, and a ResNet-50 variant, which exploits 3D convolutions.	1. Segmentation with U-Net, U-Net++, and two other networks. 2. The best results are fed to ResNets for feature extraction and classification + Data augmentation.	Both binary classification (B. vs. M. lesions) and multiclass. 10-fold cross-validation. Results are calculated over validation folds and averaged.	-	0.46 MM, 0.16 ME, 0.46 BM, 0.35 BE | 0.91 (binary	0.50 MM, 0.88 ME, 0.79 BM, 0.75 BE | 0.88 (binary)	0.98 MM, 0.83 ME, 0.90 BM, 0.93 BE | 0.89 (binary)
**Lihacova** **et al.,** **2022** [39]	MS images (1304–4 images): MM-like lesions (74), PB lesions (405), HK lesions (323), non-MMC (172), other B. lesions (330).	VIS-exNIR: 526 nm, 663 nm and 994 nm (3 spectral bands) + AF image under 405 nm.	Pre-trained InceptionV3, VGG-16 and ResNet-50. DARTS custom model trained from scratch.	1. Data augmentation. 2. Fine-tuning pre-trained models and training from scratch DARTS architecture.	5-fold cross-validation Results are calculated over validation folds and averaged.	-	-	0.72 MM, 0.83 PB, 0.61 HK, 0.57 non-MMC, 0.84 other benign (DARTS-best)	0.97 MM, 0.90 PB, 0.91 HK, 0.95 non-MMC, 0.93 other benign (DARTS-best)
**Lin et al., 2024** [41]	878 images: Acral Lentiginous Melanoma (342), Superficial Spreading Melanoma (253), Nodular Melanoma (100), Melanoma in situ (183).	SAVE: HSI synthesized from RGB. Band selection of 415 nm, 540 nm, 600 nm, 700 nm, and 780 nm;	YOLO (v5, v8, v9), SAVE algorithm integration.	1. RGB to HSI conversion using SAVE. 2. Training on augmented dataset (7:2:1 split). 3. Comparison across YOLO versions with metrics like precision, recall, mAP, and F1-score.	Training: 70%, Validation: 20%, Testing: 10%.	-	-	YOLO v8-SAVE: Precision > 90%, Recall 71%, mAP 0.801; YOLO v8-RGB: Precision > 84%, Recall 76%, Map 0.81 Superficial Spreading Melanoma: Precision decreases 7% in YOLO v5-SAVE, increases 1% in YOLO v8-SAVE.	

**Table 3:** Principal lesion etiologies in the dataset, the number of reflectance cubes, the number of reflectance images, and the identification labels.

Reflectance Cubes	ID	Diagnostic	Benign/Malignant	Reflectance Images
**112**	1	Melanoma	M	8
	1	MIS (melanoma in situ)	M	
	1	Lentigo	M	
**327**	2	Dermic Nevus	B	8
	2	Dysplastic Nevus	B	
	2	Blue Nevus	B	
**70**	3	BCC	M	8

**Table 4:** Metrics accounting for classification performance over the unseen test set for the models 2D-CNN, 3D VGG-16, 3D ResNet34, and Keras ResNet50 when trained with the full reflectance cube (414, 447, 477, 524, 671, 735, 890, and 995 nm). Acc.: accuracy; SE: sensitivity; SP: specificity; P: precision; F1: F1 score.

Model	Top 1 Acc.	SE	SP	P	F1
2D-CNN					
Nevus	0.83	0.86	0.88	0.80	0.83
Melanoma		0.79	0.96	0.92	0.85
BCC		0.86	0.88	0.86	0.86
Malignant vs. Benign	0.88	0.89	0.86	0.93	0.91
3D VGG-16					
Nevus	0.71	0.71	0.83	0.71	0.66
Melanoma		0.64	0.81	0.64	0.57
BCC		0.79	0.86	0.79	0.75
Malignant vs. Benign	0.81	0.71	0.86	0.85	0.77
3D ResNet34					
Nevus	0.74	0.79	0.77	0.65	0.71
Melanoma		0.50	1.00	1.00	0.67
BCC		0.93	0.78	0.72	0.81
Malignant vs. Benign	0.79	0.79	0.79	0.88	0.83
Keras ResNet50					
Nevus	0.55	0.64	0.67	0.56	0.60
Melanoma		0.79	0.50	0.48	0.59
BCC		0.21	1.00	1.00	0.35
Malignant vs. Benign	0.71	0.75	0.64	0.80	0.77

**Table 5:** Metrics accounting for classification performance over the unseen test set for the models 2D-CNN and 3D VGG-16 when trained with the full reflectance cube (414, 447, 477, 524, 671, 735, 890, and 995 nm) in comparison to their classification performance when trained with RGB images (447, 524, and 671 nm). Acc.: accuracy; SE: sensitivity; SP: specificity; P: precision: F1: F1 score.

Model	Top 1 Acc.	SE	SP	P	F1
2D-CNN (Reflectance Cube)					
Nevus	0.83	0.86	0.88	0.80	0.83
Melanoma		0.79	0.96	0.92	0.85
BCC		0.86	0.88	0.86	0.86
Malignant vs. Benign	0.88	0.89	0.86	0.93	0.91
2D-CNN (RGB bands)					
Nevus	0.36	0.07	0.89	0.25	0.11
Melanoma		0.93	0.18	0.36	0.52
BCC		0.07	0.96	0.50	0.13
Malignant vs. Benign	0.62	0.07	0.89	0.25	0.11
3D VGG-16 (Reflectance Cube)					
Nevus	0.71	0.71	0.83	0.71	0.66
Melanoma		0.64	0.81	0.64	0.57
BCC		0.79	0.86	0.79	0.75
Malignant vs. Benign	0.81	0.71	0.86	0.85	0.77
3D VGG-16 (RGB bands)					
Nevus	0.45	0.43	0.62	0.43	0.43
Melanoma		0.43	0.87	0.75	0.55
BCC		0.50	0.48	0.35	0.41
Malignant vs. Benign	0.62	0.43	0.71	0.43	0.43

## Data Availability

The data presented in this study are available on request from the corresponding author due to privacy reasons.

## Supplementary Materials

Pre-trained models with our MS dataset of skin cancer lesions and other supplementary data to this article can be found online at https://github.com/LauraReyBarroso93/SkinCancerCNN.

## Glossary

Multispectral Imaging (MSI)	A technique that captures image data at different wavelengths across the electromagnetic spectrum, allowing for the analysis of the spectral information of each pixel. In dermatology, MSI is used to enhance skin lesion analysis by identifying chromophore variations.
Convolutional Neural Network (CNN)	A type of Deep Learning model particularly effective for image recognition tasks. CNNs use convolutional layers to extract spatial features, making them suitable for medical image classification, such as distinguishing between types of skin lesions.
VGG-16 Model	A specific CNN architecture with 16 layers, often used in image classification. This model processes input images with high spatial resolution, making it effective for detailed medical image analysis.
Reflectance Cube	In MSI, a 3D dataset containing images captured at various spectral bands. Each ’slice’ of the cube corresponds to a different wavelength, providing detailed information on how skin reflects light at each band.
Data Augmentation	A technique to artificially increase the size of a dataset by applying transformations (such as rotation, flipping, etc.) to existing images. This helps reduce overfitting and improves model generalization, especially in small datasets.
Cross-Validation	A statistical method used to evaluate a model’s performance by dividing the dataset into multiple subsets. The model is trained on some subsets and validated on the remaining ones, improving reliability and reducing bias in performance metrics.
Spectral Bands	Specific wavelength ranges within the electromagnetic spectrum used in MSI. For skin lesion analysis, spectral bands capture the unique absorption properties of skin chromophores (e.g., melanin, hemoglobin).
Attention Maps (Saliency Maps)	Visualizations that show which areas of an image a neural network focuses on during classification. In skin lesion analysis, attention maps highlight lesion regions over the surrounding skin, validating the model’s attention.
Binary Classification	A classification task where the model assigns one of two labels to an input. In this context, binary classification refers to distinguishing between benign and malignant lesions.
Categorical Cross-Entropy (CE)	A loss function used in classification tasks with multiple classes, measuring the difference between predicted and actual class probabilities. Lower CE indicates a better-performing model.
Accuracy	A metric that represents the proportion of correctly classified instances out of the total cases. It is a primary metric in evaluating model performance for lesion classification.
Sensitivity (SE)	Also known as recall, it measures the model’s ability to correctly identify true positive cases (e.g., malignant lesions). High sensitivity is crucial in medical diagnostics to avoid missing critical cases.
Specificity (SP)	The ability of a model to correctly identify true negatives (e.g., benign lesions). High specificity reduces the number of false positives, which is important to prevent unnecessary biopsies.
Precision (P)	A measure of how many of the model’s positive predictions are actually correct. High precision indicates fewer false positives, which is valuable for accurate diagnosis.
F1 Score	The harmonic mean of precision and sensitivity, providing a balanced measure of a model’s accuracy in binary classification tasks, particularly in imbalanced datasets.
Crossed Polarizers	Optical filters used in MSI to reduce specular reflections from skin, enhancing the quality of the captured images by minimizing the glare from sweat or skin oils.
Random Grid Search	A hyperparameter tuning method where values are randomly selected within a specified range to optimize model performance without an exhaustive search.
Transfer Learning	A Deep Learning technique where a pre-trained model is fine-tuned on a new, smaller dataset. It is generally avoided in MSI with skin images due to the unique spectral characteristics of these data.
Learning Rate (LR)	A hyperparameter in Deep Learning that controls the step size at each iteration of optimization. The learning rate is crucial for model convergence and stability.
